# Mechanical Behavior of Bio-Inspired Honeycomb–Core Composite Sandwich Structures to Low-Velocity Dynamic Loading

**DOI:** 10.3390/ma17051191

**Published:** 2024-03-04

**Authors:** Tao Tao, Lizheng Li, Qiang He, Yonghui Wang, Junlan Guo

**Affiliations:** 1 Guangzhou Metro Design & Research Institute Co., Ltd., Guangzhou 510010, China; taotao@gmdi.cn; 2 School of Mechanical Engineering, Jiangsu University of Science and Technology, Zhenjiang 212000, Chinajunlan0815@163.com (J.G.)

**Keywords:** sandwich structure, low-velocity impact, numerical analysis, energy absorption

## Abstract

In order to improve the impact resistance of sandwich panels under low-velocity impact, the lotus leaf vein is selected as a biological prototype to design a bio-inspired honeycomb (BIH) sandwich panel. ABAQUS is used to establish and effectively verify the finite element (FE) model of the BIH sandwich panel. To systematically compare and study the mechanical properties of BIH and conventional hexagonal honeycomb sandwich panels under low-velocity impact, the maximum displacement of face-sheets, the deformation mode, the plastic energy consumption and the dynamic response curve of the impact end are presented. At the same time, the performance differences between them are revealed from the perspective of an energy absorption mechanism. Furthermore, the influence of the circumscribed circle diameter ratio of the BIH trunk to branch (*γ*), the thickness ratio of the trunk to branch (*K*) and the impact angle (*θ*) on impact resistance is studied. Finally, the BIH sandwich panel is further optimized by using the response surface method. It can be concluded that, compared to conventional hexagonal honeycomb sandwich panels, the addition of walls in the BIH sandwich panel reduces the maximum deformation of the rear face-sheet by 10.29% and increases plastic energy consumption by 8.02%. Properly adjusting the structural parameters can effectively enhance the impact resistance of the BIH sandwich panel.

## 1. Introduction

As a typical lightweight thin-walled structure, honeycomb sandwich panels have been widely used as protective equipment due to their outstanding impact resistance and meeting the requirements of lightweight design [1,2,3]. They also have a wide range of applications in the fields of aviation [4], automobiles [5] and personal protective equipment [6]. Therefore, the honeycomb sandwich structure has always been a hot topic for scholars [7,8,9,10,11,12]. Rejab [13] et al. focused on studying the mechanical properties of corrugated sandwich panels, which exhibit stronger impact resistance. Sun [14] et al. introduced periodic grids to enhance the hexagonal honeycomb core sandwich structure and conducted plane compression experiments and numerical studies. Research has shown that the mechanical properties of sandwich panels were closely related to their core structure. Lan [15] et al. comprehensively analyzed the dynamic response of hexagonal honeycomb cores and cylindrical sandwich panels with auxetic concave honeycomb cores. The cylindrical sandwich panel with auxetic concave honeycomb cores was proven to have a better anti-explosive performance than those with hexagonal honeycomb cores. McShane et al. [16] found that the three special-shaped sandwich panels have better impact resistance.

Meanwhile, the honeycomb sandwich structure is very vulnerable to low-speed impacts from gravel, hail, etc. Therefore, scholars have paid great attention to the mechanical properties of honeycomb sandwich panels under low-speed impact. Sun et al. [17] analyzed the low-velocity impact characteristics of sandwich panels with different structural parameters and carried out relevant experimental research. Zhang et al. [18,19] combined experimental and numerical methods to derive an analytical model of energy balance based on the dynamic response of a spherical indenter impacting the sandwich panel at low speed. The research of Liu et al. [20] showed that sandwich panels with round tube-filled hexagonal honeycomb cores had better impact resistance than conventional hexagonal honeycomb core sandwich panels. Reyes and Børvik [21] conducted quasi-static and dynamic experiments on the impact resistance of polymer foam core sandwich panels. Zhou [22] proposed an auxetic recessed honeycomb filled with new foam concrete and found that, with an increase in foam concrete density, the structure changed from a compression failure mode to a shear failure mode. Dhanarasu et al. [23] systematically analyzed the mechanical response of sandwich panels with CFRP face-sheets. Dai and Akatay [24,25] focused on the effects of repeated impacts on sandwich panels. Zhang and Al [26,27] proposed tube-reinforced honeycomb and comprehensively analyzed its mechanical properties.

Numerous studies have shown that the performance of sandwich plates is closely related to its cell structure [28,29,30,31,32,33]. With the improvement in energy absorption capacity requirements, how to design a honeycomb sandwich structure with better impact resistance has been a challenge for scholars. Researchers have found that applying biological structural features to the design of thin-walled structures can effectively improve their crashworthiness [34,35,36,37,38]. Song et al. [39] designed a new type of bionic tube with grooves and studied its crashworthiness under lateral impact. They found that the energy absorption efficiency of the bionic tube was better than that of the circular tube. Based on the structural characteristics of bamboo, Zou et al. [40] proposed a bionic tube and solved its numerical examples under axial/transverse impact. The results showed that the bionic design improved the specific energy absorption of the tube. Palombini et al. [41] mechanically explored the special geometry of a single vascular bundle in bamboo, and the new design had a noticeable improvement in its strength and energy absorption under axial/transverse loads. It is widely known that the leaf veins not only carry nutrients, but also support the weight and external load of the whole blade structure to ensure the overall stiffness and plane ductility of the blade. He et al. [42] introduced this leaf-vein-branched characteristic into the honeycomb in previous studies, but they only focused on the dynamic response under in-plane dynamic compression.

Inspired by the lotus leaf vein biological structure, we designed a bio-inspired honeycomb (BIH) core sandwich structure. Based on ABAQUS 6.14 software, the dynamic response process of the BIH sandwich panel under low-speed dynamic impact was systematically investigated. Firstly, the reasonableness and accuracy of the simulation method used in this paper were verified by comparing them with previous experimental results. Secondly, the dynamic responses of the BIH sandwich panel and conventional hexagonal honeycomb sandwich panels under low-velocity impact were systematically compared, and the performance difference between them was analyzed from the perspective of the energy absorption mechanism. Then, the coupling influence law of the structural parameters of the BIH sandwich panel and its mechanical properties was further analyzed by parametric study. Finally, the BIH sandwich structure was optimized using the response surface method.

## 2. Geometric Model of the BIH Sandwich Structure

**Figure 1:**
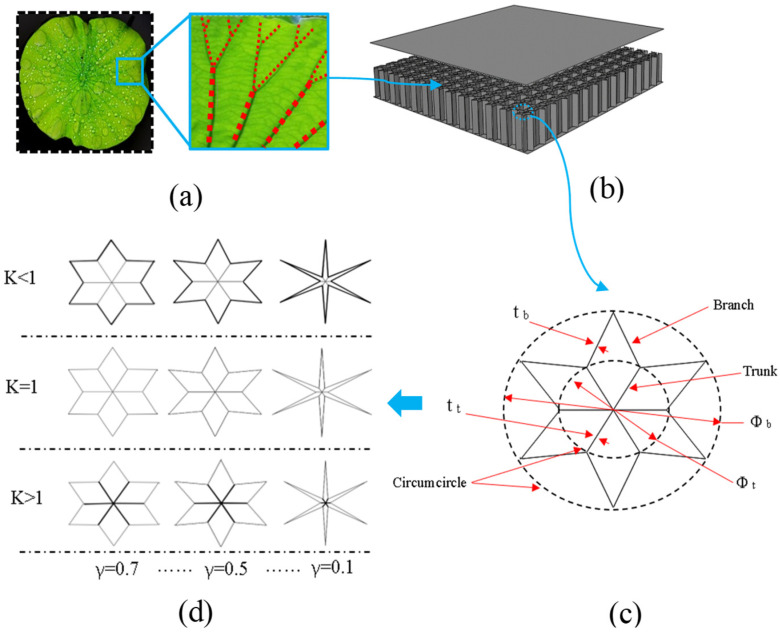
The design process and geometric configuration of the bio-inspired honeycomb sandwich structure: (**a**) geometry of lotus leaf veins; (**b**) BIH core sandwich structure; (**c**) cell geometry; (**d**) cross-sectional evolution of BIH cells.

Inspired by the biological structure of nature, the lotus leaf vein was chosen as a biological prototype. Figure 1 shows the design process and geometric configuration of the bio-inspired honeycomb core sandwich structure. The geometric structure of the lotus leaf vein in Figure 1a is characterized by the main trunk branch, and the cross-sectional size of the trunks is larger than that of the branches. Figure 1b shows the BIH core sandwich structure and its cell geometry is shown in Figure 1c. The dotted circles, as seen in the picture, are the circumscribed circles of the main trunks and branches. *t_t_* and *t_b_* are the wall thicknesses of the trunks and branches, respectively, and *Φ_b_* and *Φ_t_* are the circumscribed circle diameter of the branches and trunks, respectively. The BIH structure parameter, *K*, is the ratio of *t_t_* to *t_b_*, and *γ* is the ratio of *Φ_t_* to *Φ_b_*, as shown in Equations (1) and (2). The branch circumscribed circle diameter *Φ_b_* is 12 mm, and the sum of *t_t_* and *t_b_* is 0.15 mm, as shown in Equations (3) and (4). The range of values for *γ* and *K* are 0.1 to 0.7 and 0 to 10, respectively. The structure size and wall thickness of the BIH change with parameters *K* and *γ*, respectively. Figure 1d depicts the cross-sectional evolution of BIH cells, and Table 1 gives the specific parameters of the BIH sandwich panel.

(1) $$K=\frac{t_{t}}{t_{b}}\;0<K<10$$

(2) $$\gamma =\frac{\Phi_{t}}{\Phi_{b}}\;0.1\leq \gamma \leq 0.7$$

(3) $$t_{t}+t_{b}=0.15\;mm$$

(4) $$\Phi_{b}=12\;mm$$

## 3. Finite Element Method of BIH Sandwich Structure

### 3.1. FE Model

**Figure 2:**
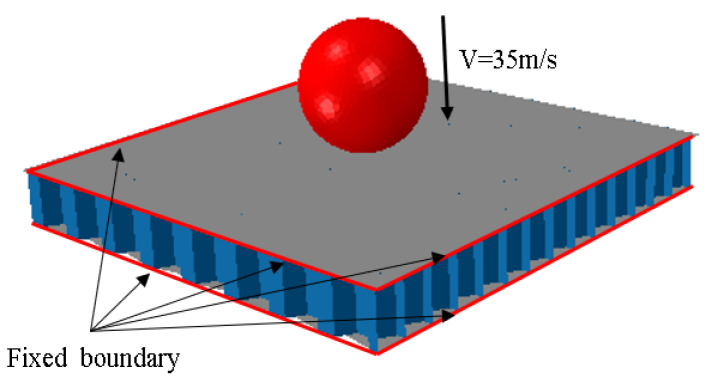
FE model of BIH under low-velocity impact.

ABAQUS is used to simulate the mechanical properties of the BIH sandwich panel under low-speed impact, and the numerical simulation model is depicted in Figure 2. The material model of the BIH sandwich structure adopts a bilinear elastoplastic model, the panel material is AL5083 and the honeycomb core material is AL3003 [18]. The specific material parameters of AL5083 and AL3003 are shown in Table 2. A binding constraint is adopted between the honeycomb core and the panel; the trunk and branches are connected together by means of co-nodes. The diameter and mass of the spherical impactor are 40 mm and 0.2 kg, respectively. The mass and velocity are set at the center of the sphere and the impact direction is downward and perpendicular to the sandwich panel. The impact point is at the center of the sandwich panel. When the spherical impactor impacts the BIH interlayer structure, the speed of the spherical impactor is 35 m/s [20]. The spherical impact block is set as a rigid body and meshed by 4-node 3D bilinear rigid elements. The 4-node doubly curved shell element is used to mesh the face-sheets and the honeycomb core. Meanwhile, the contact between the spherical impactor and the sandwich panel is simulated by a surface-to-surface contact. A general contact is set for the rest of the contacts to avoid interpenetration of the honeycomb cores. The static and dynamic friction coefficients between each structure are both set to 0.3 [20]. The three sides of the face-sheets are fixed [18], as reflected in the red line in Figure 2.

**Figure 3:**
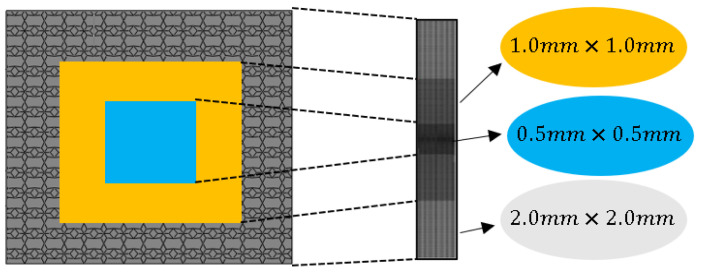
Meshing of the finite element model.

The grid size of the finite element model has an inseparable influence on the calculation cost and accuracy. After weighing the calculation cost and accuracy, a gradient grid is used to divide the finite element model into several areas. According to Figure 3, the light blue area is meshed by a grid with a size of 0.5 mm × 0.5 mm, the grid size of the orange area is 1.0 mm × 1.0 mm and the other areas are 2.0 mm × 2.0 mm. 

### 3.2. Validity Verification

**Figure 4:**
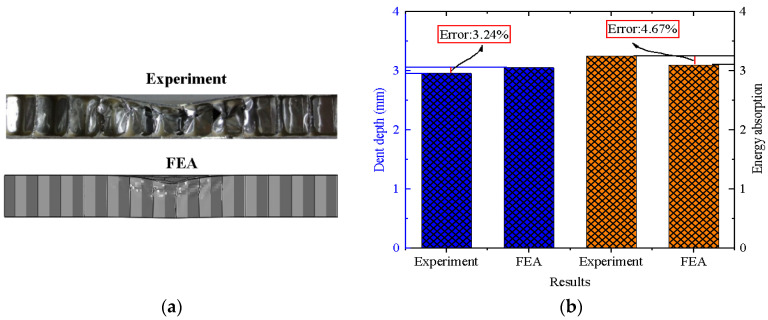
Comparison of experimental [18] and numerical results: (**a**) deformation mode; (**b**) dent depth and energy absorption.

The experimental results of Zhang [18] were used to verify the validity of the FE model. The detailed information of drop-weight tests was reported in [18]. In order to be consistent with the test conditions, the weight of the impactor was 0.3 kg with a radius of 20 mm and an impact velocity of 5.05 m/s. Other relevant parameters and settings, such as the structural dimensions, the material properties and the boundary conditions, are consistent with those in Section 3.1. The numerical simulation results were compared with the experimental results, as shown in Figure 4. It can be seen from the map that the experimental and numerical deformation modes are in good agreement. The errors of the dent depth on the rear face-sheet and the energy absorption are 3.24% and 4.67%, respectively. Therefore, the FE method and the material model are accurate enough to be used to simulate the dynamic response of the BIH sandwich panels.

## 4. Comparative Analysis of Two Honeycomb Core Sandwich Structures

### 4.1. Dynamic Response Analysis

**Figure 5:**
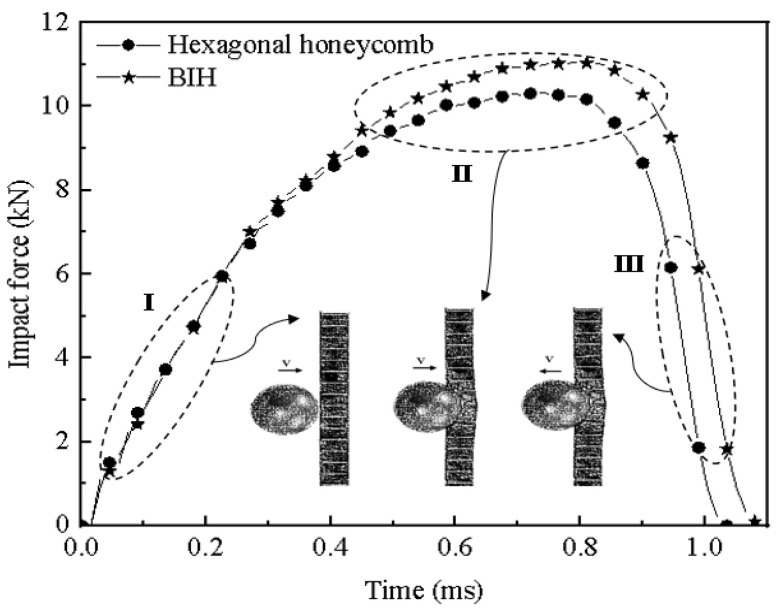
Comparison of time history curves of impact load of sandwich structure.

In order to compare the performance difference between the BIH and the conventional hexagonal honeycomb sandwich panel, the cell wall thickness of the honeycomb core is adjusted to ensure the same mass. The sizes of both honeycomb cores are 150 mm×150 mm×16 mm. The other structural parameters are shown in Table 3. Figure 5 shows the dynamic response curve at the impact end. The dynamic response of the impact end of these two structures shows a trend of first increasing and then decreasing. When the spherical block impacts the two sandwich structures, it goes through three stages, namely, an elastic stage (I), a plastic deformation stage (II) and a rebound stage (III). In the elastic phase, the BIH sandwich structure has the same impact level as the conventional hexagonal honeycomb sandwich panel, which shows that the initial structural rigidity of the BIH sandwich panel is similar to that of the conventional structure. In the plastic deformation stage, the impact load level of the BIH sandwich structure is higher than that of the conventional hexagonal honeycomb sandwich structure.

**Figure 6:**
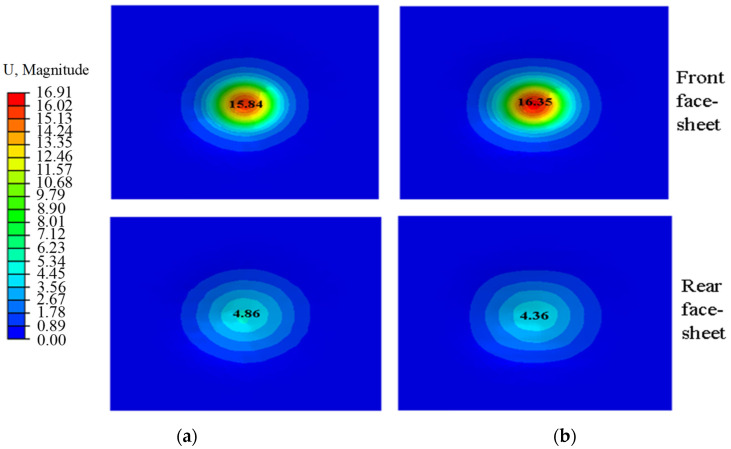
Comparison of maximum displacement of sandwich structure face-sheets: (**a**) conventional hexagonal honeycomb sandwich structure; (**b**) BIH sandwich structure.

Figure 6 exhibits the maximum displacement of the face-sheet of the BIH sandwich structure and the conventional hexagonal honeycomb sandwich structure. The maximum displacement of the front face-sheet of the BIH sandwich structure is 16.35 mm, which is 3.22% higher than the 15.84 mm conventional sandwich structure displacement. However, the maximum displacement of the rear panel decreased by 10.29% compared to the conventional sandwich structure. This means that the BIH sandwich cores are easier to compress and deform than the conventional hexagonal honeycomb sandwich cores, which is beneficial for the absorption of more impact kinetic energy. Therefore, the BIH sandwich structure shows better impact resistance.

### 4.2. Energy Absorption and Its Enhancement Mechanism

**Figure 7:**
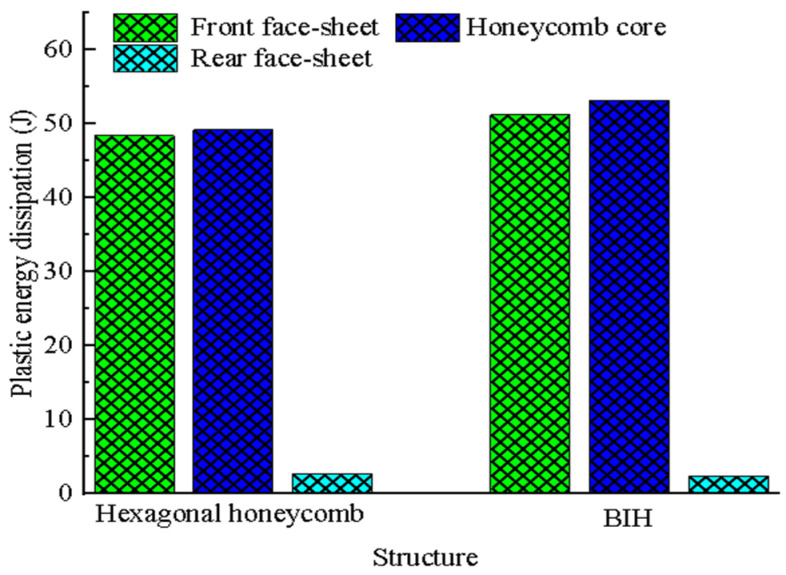
Comparison of plastic energy consumption of the hexagonal honeycomb and BIH sandwich structures.

Figure 7 exhibits the plastic energy consumption of each part of the BIH sandwich structure and the conventional hexagonal honeycomb sandwich structure. The plastic energy consumptions of the front face-sheet and the honeycomb core of the BIH sandwich panel are higher than those of the conventional hexagonal honeycomb sandwich panel. The plastic energy consumption of the rear face-sheet of the BIH sandwich panel is lower than that of the conventional panel. Among them, the plastic energy consumption of the BIH is 8.02% higher than that of the hexagonal honeycomb. The damage to the rear face-sheet may be reduced considering that the front face-sheet and the honeycomb core of the BIH sandwich panel absorb most of the impact energy. 

**Figure 8:**
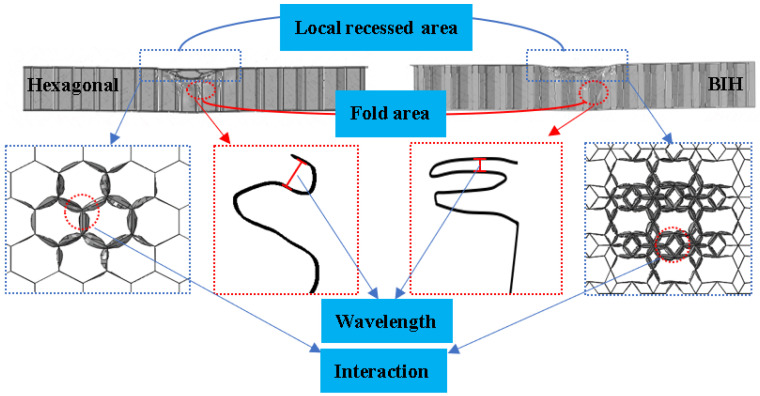
Deformation mode of sandwich structures with partially enlarged areas.

The deformation modes are given in Figure 8. The interaction between honeycomb cells mainly occurs near the local recessed area, and the buckling of the cells occurs in the fold area. Evidently, the interaction enhancement effect between the BIH cells is more obvious than that of the conventional hexagonal honeycomb, and the folding wavelength of the BIH fold area is smaller than that of the hexagonal honeycomb. Generally, the more interactions between honeycomb cells, the better the impact energy dissipation. The shorter folded wavelengths are beneficial to the formation of more folds, which causes more energy to be absorbed. This analysis explains why the BIH sandwich structure has better impact resistance than the conventional hexagonal honeycomb sandwich structure.

## 5. Parameter Study

In order to reveal the coupling effects of the structural parameters and their impact resistance, the influence of the circumscribed circle diameter ratio of the BIH trunks and branches (*γ*), the thickness ratio of trunks to branches (*K*) and the impact angle (*θ*) were investigated.

### 5.1. Effect of Ratio γ 

**Figure 9:**
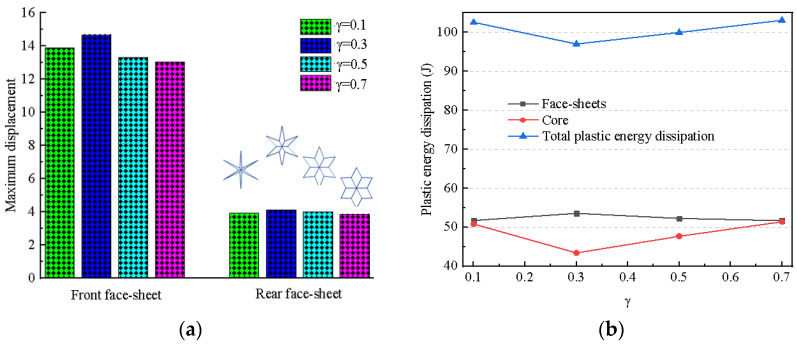
Maximum displacement and plastic energy dissipation of BIH sandwich structure with different *γ* ratios: (**a**) maximum displacement; (**b**) plastic energy dissipation.

Four different *γ* values were designed as follows: 0.1, 0.3, 0.5, 0.7. At the same time, the parameter *K* remained unchanged (*K* = 1). Figure 9 shows the maximum displacement and the plastic energy dissipation of the BIH sandwich structure with different *γ* ratios. The maximum displacements of front and rear face-sheets both increase and then decrease as *γ* increases. In particular, when *γ* is equal to 0.3, the front and rear face-sheets have the largest displacement. Figure 9b describes the plastic energy dissipation of the BIH sandwich structure. As *γ* increases, the plastic energy dissipation of the whole structure and the honeycomb core decreases and then increases, while the plastic energy dissipation law of the structural face-sheets is exactly the opposite to the previous two. It should be noted that when *γ* is 0.3, the plastic energy dissipation values of the overall structure and the honeycomb core are the lowest, while the plastic energy dissipation value of the face-sheet is the highest. This means that the honeycomb core has important impacts on its total plastic dissipated energy, which may further determine the impact resistance performance of this structure.

**Figure 10:**
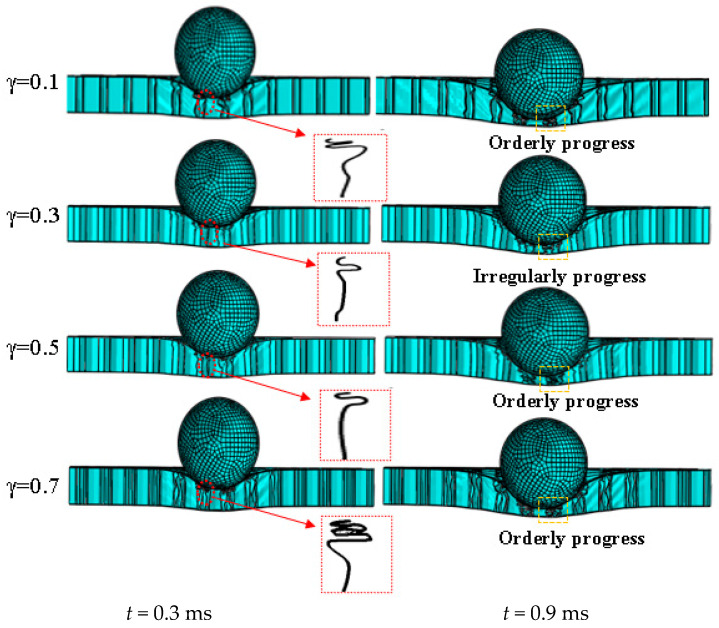
Deformation mode of BIH sandwich structure under different *γ* ratios.

Therefore, Figure 10 exhibits the deformation modes of the BIH sandwich structure at 0.3 ms and 0.9 ms under different *γ* values. When the BIH sandwich structure is impacted by a solid sphere at low speed, the central area is in a depression state and the cell walls in this region buckle plastically. The contour line of cell buckling is enlarged inside the red dashed box. When *γ* is equal to 0.7, the folding wavelength is the shortest, followed by *γ* equal to 0.1. Interestingly, the structures with *γ =* 0.3 and *γ =* 0.5 have similar folding wavelengths. According to the plastic collapse of the cells in the orange dotted box, it can be clearly seen that the cell walls of the sandwich structure, with *γ =* 0.1, *γ =* 0.5 and *γ =* 0.7, gradually fold in an orderly manner, which is different from the irregular folding in the sandwich structure with *γ =* 0.3. Ordered cell folding means that more impact energy is effectively absorbed. Therefore, when designing the BIH sandwich energy-absorbing structure, it should be noted that *γ* equal to 0.3 is not conducive to impact resistance.

### 5.2. Effect of Ratio K

**Figure 11:**
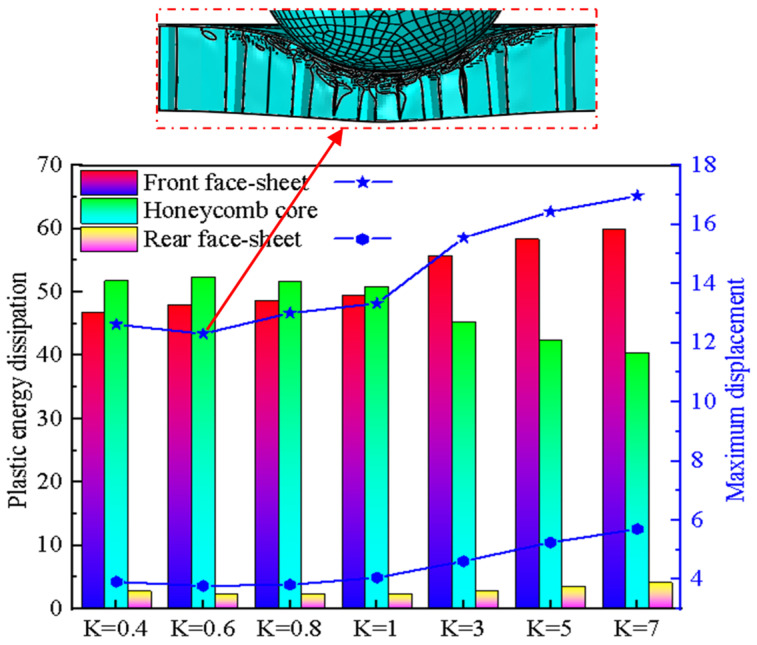
The maximum displacement and plastic energy dissipation of BIH sandwich structure under different values of ratio *K*.

To analyze the influence of the *K* ratio on the dynamic response of the BIH sandwich panel, seven different *K* values were designed as follows: 0.4, 0.6, 0.8, 1, 3, 5, 7. At the same time, the parameter *γ* remained unchanged (*γ* = 0.5). Figure 11 shows the maximum displacement and the plastic energy dissipation of the BIH sandwich structure with different values of *K*. When *K* ≤ 1, the plastic energy consumption of the face-sheets and the honeycomb core of the BIH sandwich structure changes very little with the change in *K*. However, when *K* > 1, they all have significant differences with the change in *K*. The maximum displacement of both the front and rear face-sheets decreases and then increases with the increase in *K*. In particular, the rear face-sheet of the BIH sandwich structure has a minimum depth of depression when *K* = 0.6. This means that when *K* = 0.6, the BIH sandwich structure has the best impact resistance, and its deformation mode is shown in the red dashed box of the picture.

### 5.3. Effect of Impact Angle θ

**Figure 12:**
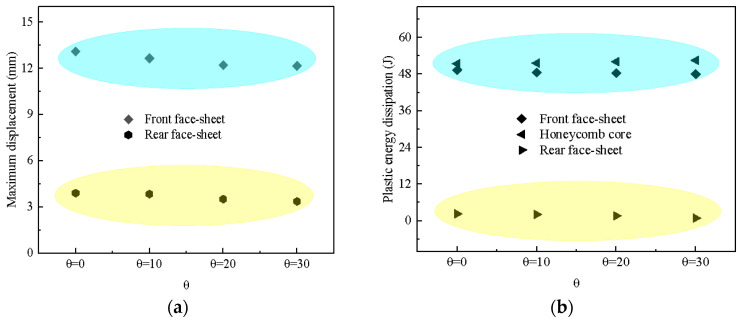
Maximum displacement and plastic energy dissipation of BIH sandwich structure under different values of *θ*: (**a**) maximum displacement; (**b**) plastic energy dissipation.

Considering that sandwich panels may be subjected to impact loads from different directions, an analysis of the impact angle *θ* is of great significance. Four different *θ* values were designed as follows: 0°, 10°, 20°, 30°. At the same time, *K* and *γ* were set to 1 and 0.5, respectively. Figure 12 depicts the maximum displacement and the plastic energy dissipation of the BIH sandwich structure at different impact angles. The maximum displacement of the front and rear face-sheets gradually decreases as *θ* increases. In Figure 12b, the plastic energy dissipation of the front and rear face-sheets of the BIH sandwich panel gradually decreases with the increase in *θ*. However, the plastic dissipated energy of the BIH core is the opposite. The impact from different angles will cause varying degrees of damage to the BIH sandwich panel.

**Figure 13:**
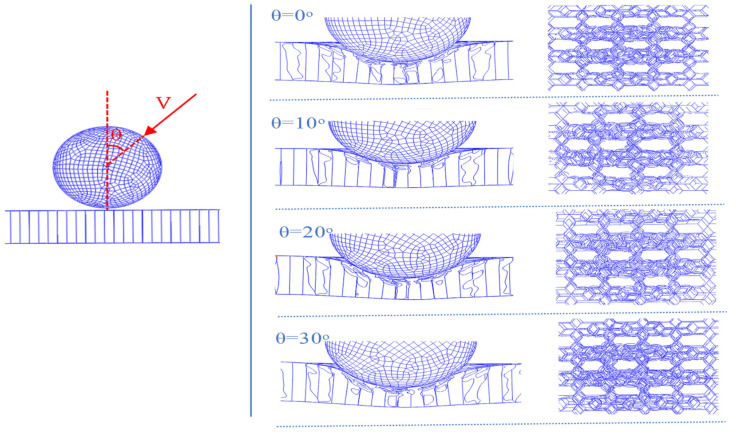
Deformation mode of BIH sandwich structure under different values of *θ*.

Figure 13 gives the deformation mode of the BIH sandwich structure for different values of *θ*. As the impact angle increases, the number of wrinkles at the local buckling of the sandwich structure increases. In addition, the interaction between the cells of the BIH gradually increases. Therefore, it can be concluded that the energy absorption characteristics of the structure are closely related to the impact angle.

## 6. Optimization Design

### 6.1. Determination of Optimization Problem

In order to obtain the BIH sandwich panel with the best impact resistance, the structural parameters of the BIH sandwich structure were further optimized. In the process of design optimization, the weight efficiency control of the BIH sandwich structure cannot be ignored. The dent depth and the mass of the BIH sandwich structure are represented by *U* and *M*, respectively. The response surface method [43,44,45] is used to optimize the design of the BIH sandwich panel. The expression of the optimization problem can be described as follows:

(5) $$\left\{\begin{array}{c} \begin{array}{c} Minimize\;[U(K,\gamma )]\\ 0.4\leq K\leq 1.7 \end{array}\\ \begin{array}{c} 0.1\leq \gamma \leq 0.7\\ \;[M(K,\gamma )]\leq 100\;g \end{array} \end{array}\right.$$

### 6.2. Response Surface Method

#### 6.2.1. The Process of Design Optimization

**Figure 14:**
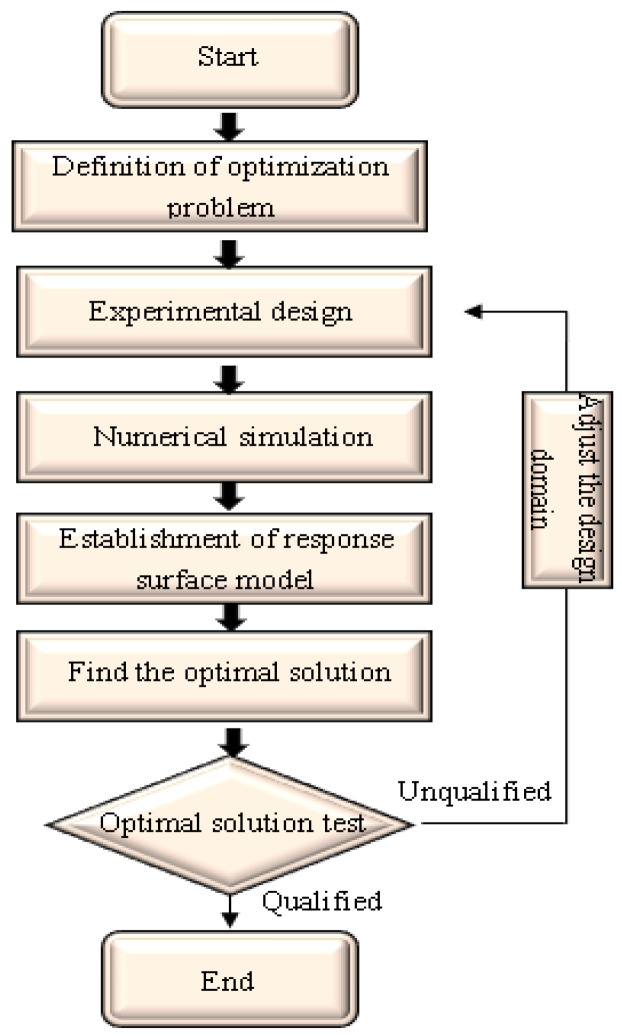
Optimization process of response surface method.

The optimization design process using the response surface method is shown in Figure 14. The optimization problem is first determined. Then, the design of experiments is carried out according to the optimization problem to obtain the corresponding design sample points. The corresponding value of the objective function is obtained through finite element simulation. Subsequently, a response surface model is established through regression analysis based on the design sample points and their response values, and the optimal solution is then obtained from the response surface model. Finally, the optimal solution is tested. If the error between the optimal result obtained from the prediction model and the numerical simulation result is within the allowable range, the optimal solution is considered acceptable. If the error between them is large, the design domain needs to be adjusted, re-optimized and analyzed until the error is within the allowable range.

#### 6.2.2. Design of Experiments and Metamodel

**Figure 15:**
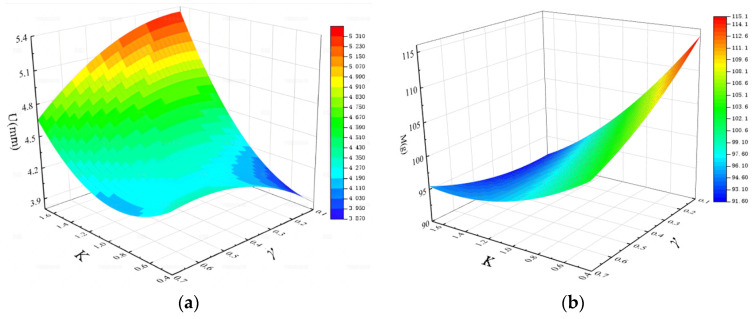
(**a**) The response surface of *U*; (**b**) the response surface of *M*.

Typical design experiments include the Taguchi orthogonal experiment method [46,47], the central composite design, the full factor design and the Latin hypercube. The full factor design was adopted to generate 20 design sampling points (four levels for *K*, five levels for *γ*), and the response values of the selected sample points were obtained by simulation. Polynomial regression analysis was used to deal with the design optimization problems [48,49]. The least squares method was used to fit *U* and *M*. Figure 15 is the response surface and the predictive models of *U* and *M* are as follows:

(6) $$U=3.698-0.430K+2.957\gamma -1.485K\gamma +0.817K^{2}-1.984\gamma^{2}$$

(7) $$M=130.298-32.632K-41.814\gamma +21.091K\gamma +6.007K^{2}+15.25\gamma^{2}$$

**Figure 16:**
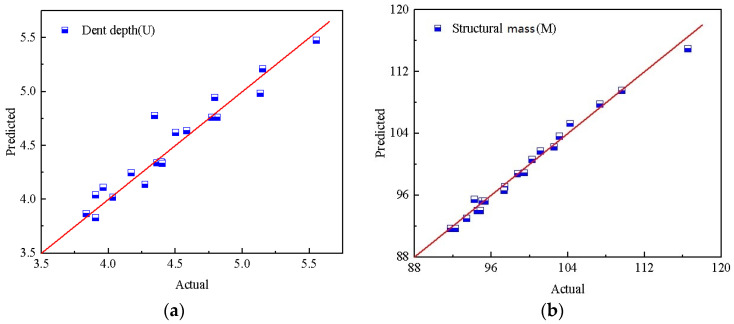
Comparison of predicted and actual results: (**a**) dent depth, *U*; (**b**) structural mass, *M*.

Figure 16 compares the predicted value of the response surface model with the actual value. It can be seen that all the square dots fluctuate around the red diagonal line with a slope of 1. The predicted values of *U* and *M* are not significantly different from the actual values, which further verifies the accuracy of the response surface model.

### 6.3. Results and Discussion

**Figure 17:**
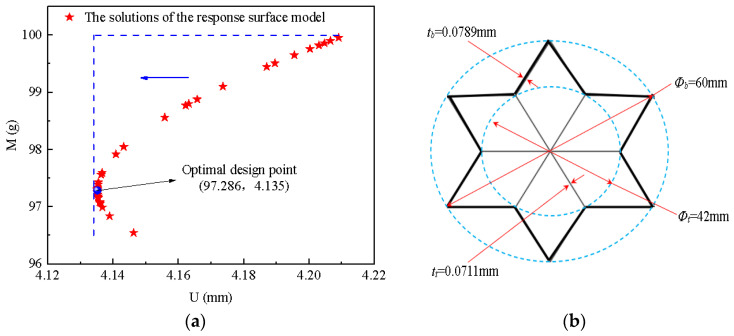
(**a**) The solutions of the response surface model; (**b**) optimal BIH cell cross section.

In order to obtain the optimal BIH sandwich structure, Figure 17a shows a partial solution of the response surface model during the optimization process. The blue dot is the optimal design point to obtain the smallest *U* value, and its detailed cross-sectional parameters are given in Figure 17b. Interestingly, when the mass is less than 97.286 g, the dent depth of the rear face-sheet increases as the mass increases. However, when the mass is greater than 97.286 g, the dent depth of the rear face-sheet decreases as the mass increases. This shows that when the mass is greater than 97.286 g, reducing the mass of the BIH sandwich structure is conducive to the enhancement of its impact resistance. This is of great benefit in engineering applications.

The optimal solution of the response surface model is *U* = 4.135 mm, mass *M* = 97.286 g, and the corresponding ratios are *K* = 0.9 and *γ* = 0.7. The BIH sandwich structure with the same structural parameters must be simulated to further verify the credibility of the optimal result. The simulation value of *U* is 4.246 mm, and the error between prediction and simulation is 2.61% (Table 4), which is within an acceptable range. Therefore, the optimal solution obtained by the response surface model is highly reliable. The maximum rear face-sheet displacement *U* was used as the evaluation index for the sandwich panel’s impact resistance. It can be concluded that the impact resistance of the optimized sandwich panel increased by 5.16% compared to the un-optimized sandwich panel.

## 7. Conclusions

Inspired by the vein structure of a lotus leaf, a bio-inspired honeycomb (BIH) core sandwich structure was proposed to further enhance the impact resistance of conventional honeycomb sandwich structures. ABAQUS was used to establish and effectively verify the numerical model of the BIH sandwich structure. Then, the dynamic responses of the BIH sandwich panel and the conventional hexagonal honeycomb sandwich panel under low-velocity impact were compared and analyzed, followed by a detailed analysis of the coupling effect of structural parameters and impact resistance. Finally, the BIH sandwich panel was optimized using the response surface method.

Considering that the BIH is more prone to plastic deformation and there is more contact between cells, it may be concluded that the BIH has better impact resistance than conventional hexagonal honeycomb sandwich panels. Meanwhile, the impact resistance of the sandwich panel decreased and then increased as the circumscribed circle diameter ratio of the BIH trunk to branch (*γ*) increased; the sandwich panel with *γ* = 0.3 showed the worst impact resistance. However, the thickness ratio of trunk to branch (*K*) showed the opposite trend. The impact resistance increased and then decreased as K increased and the sandwich panel with *K* = 0.6 showed the best performance. As for the influence of the impact angle (*θ*), the impact resistance of the sandwich panel gradually increased as *θ* increased. The coupling effect between the structural parameters and the energy absorption capacity can provide a reference for design optimization and engineering applications.

Based on the sample points obtained by the full-factor design of the experiments and their response values, the prediction models of *U* and *M* were obtained by polynomial regression analysis. The optimization results were further tested, which verified the reliability of the optimization results obtained from the response surface model. These results can provide valuable suggestions for the research and design of new honeycomb sandwich structures.

## Figures and Tables

**Table 1:** Structural parameters of the BIH sandwich panel.

Face-Sheets (AL5083)		BIH Core (AL3003)	
Dimension	Thickness	*K*	*γ*
150 mm×150 mm×16 mm	0.5 mm	0.4; 0.6; 0.8; 1; 3; 5; 7	0.1; 0.2; 0.3; 0.4; 0.5; 0.6; 0.7

**Table 2:** Material parameters [18].

	$\rho \;(kg/m^{3})$	E (GPa)	v	${Yield\;Stress\;\sigma }_{y}$ (MPa)	${Tangent\;Modulus\;E}_{t}$ (MPa)
AL5083 (face-sheets)	2700	72	0.33	280	933
AL3003 (BIH core)	2700	70	0.33	185	720

**Table 3:** Structural parameters of the honeycomb core.

Hexagonal Honeycomb Core		BIH Core			
Cell wall length	Wall thickness	*Φ_b_*	*t_b_*	*K*	*γ*
6 mm	0.193 mm	12 mm	0.075 mm	1	0.5

**Table 4:** Comparison of optimal solution and numerical result.

	Optimal Solution	FEM	Error
*U* (mm)	4.135	4.246	2.61%
*M* (g)	97.286	98	0.729%

## Data Availability

Data are contained within the article.
